# Crystal structure of aqua-*trans*-bis­(dimethyl sulfoxide-κ*O*)(pyridine-2,6-di­carboxyl­ato-κ^3^ *O* ^2^,*N*,*O* ^6^)nickel(II)

**DOI:** 10.1107/S2056989017006090

**Published:** 2017-04-28

**Authors:** Chen Liu, Ashley C. Felts, Daisuke Takahashi, Wesley S. Kinden, Khalil A. Abboud

**Affiliations:** a Department of Chemistry and Environmental Science, Grenfell Campus, Memorial University of Newfoundland, Corner Brook, NL, A2H 5G4, Canada; b Department of Chemistry, University of Florida, Gainesville, FL, 32611-7200, USA

**Keywords:** crystal structure, pyridine-2,6-di­carb­oxy­lic acid dianion, dimethyl sulfoxide, nickel(II), hydrogen bonding

## Abstract

In the title complex, [Ni(C_7_H_3_NO_4_)(C_2_H_6_OS)_2_(H_2_O)], the Ni^II^ cation is situated on a twofold rotation axis and exhibits a distorted octa­hedral NO_5_ coordination environment defined by a tridentate pyridine-2,6-di­carb­oxy­lic acid dianion (dpa^2−^), two dimethyl sulfoxide (DMSO) mol­ecules, and a water mol­ecule. In the crystal, the complex mol­ecules are linked by O—H⋯O and C—H⋯O hydrogen bonds into a three-dimensional network whereby DMSO mol­ecules from neighboring complexes overlap to form layers parallel to (001), alternating with layers of Ni^II^–dpa^2−^ moieties. The title complex is isotypic with its cobalt(II) analogue.

## Abstract 2

The title complex is situated on a twofold rotation axis and forms an alternating layered structure with a a three-dimensional hydrogen-bonding network.

## Chemical context   

Crystal engineering plays an important role in the research of mol­ecule-based functional materials by providing an effective approach towards the rational design and preparation of compounds with special structural features (Robin & Fromm, 2006 ▸; Cook *et al.*, 2013 ▸; Wang *et al.*, 2013 ▸). The crystallization of coordination polymers involves both the formation of a local coordination geometry and the propagation and packing of extended polymeric structures in the three-dimensional space. The competition among various types of inter­molecular inter­actions plays a critical role in this process and is strongly influenced by synthetic conditions such as the choice of solvent, temperature, and the mol­ecular features of the starting materials (Li & Du, 2011 ▸; Du *et al.*, 2013 ▸). Although much has been learned about how the synthetic conditions affect inter­molecular inter­actions and the final crystal structures, the targeted synthesis of a coordination polymer with a particular crystal structure is still a challenge. We recently reported an Ni^II^-containing one-dimensional coordination polymer based on the tridentate 2,6-pyridine di­carb­oxy­lic acid dianion (dpa^2−^) and bridging pyrazine mol­ecules that was prepared by using DMSO as the solvent (Liu *et al.*, 2016 ▸). The one-dimensional polymeric structure exhibits π–π inter­actions that were not previously observed when water was used as the solvent under the same preparation conditions. In order to explore the bridging effect of substituted pyrazine, we have repeated this preparation using 2-chloro­pyrazine to replace pyrazine under the same synthetic conditions. We report herein the synthesis and crystal structure of the resulting title compound for which incorporation of 2-chloro­pyrazine was not observed.
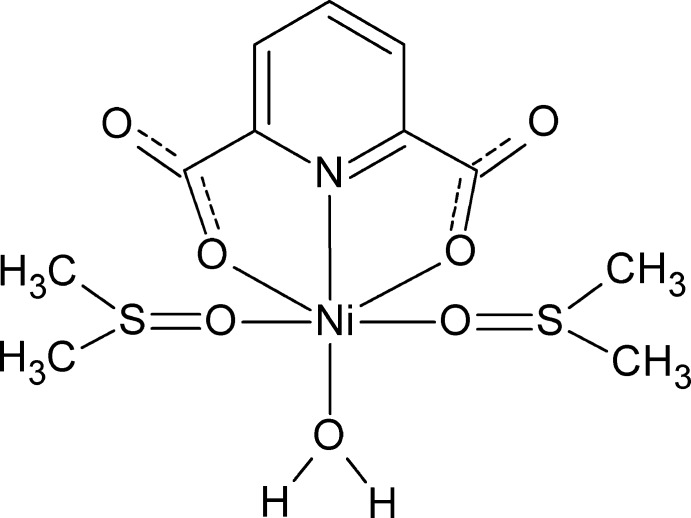


## Structural commentary   

**Figure 1:**
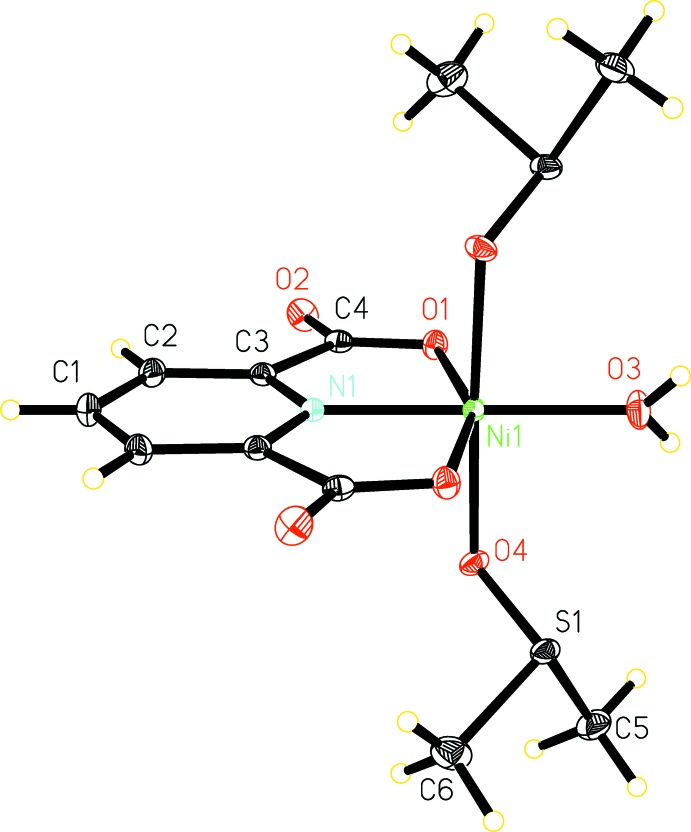
The mol­ecular structure of the title compound, with displacement ellipsoids drawn at the 50% probability level. Unlabeled atoms are related by the symmetry transformation −*x*, *y*, 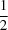 − *z*.

The title complex crystallizes in the monoclinic space group *C*2/*c* with half of the mol­ecule in the asymmetric unit, the other half being generated by twofold rotation symmetry. The tridentate 2,6-pyridine di­carb­oxy­lic acid dianion coordinates to the Ni^II^ cation in a meridional fashion *via* the pyridine nitro­gen atom and two carboxyl­ate oxygen atoms (Fig. 1 ▸). The reactant 2-chloro­pyrazine is not found in the structure of the title complex. Instead, the Ni^II^ cation is further coordinated by two *trans*-positioned DMSO mol­ecules and a water mol­ecule through their oxygen atoms. Water mol­ecules may have been produced as a result of the reaction between 2,6-pyridine di­carb­oxy­lic acid and nickel carbonate. The two Ni1—O1_dpa_ bonds have the same length 2.1130 (7) Å] and the two Ni1—O4_DMSO_ bonds have the same length [2.0934 (7) Å]. The Ni1—N1 bond length is 1.9613 (12) Å and the Ni1—O3_water_ bond length is 2.0040 (11) Å, both being significantly shorter than the other four bonds, resulting in a distorted octa­hedral NO_5_ coordination environment of the Ni^II^ cation. These bond lengths are very similar to those observed in the pyrazine-bridged one-dimensional structure reported previously (Liu *et al.*, 2016 ▸).

## Supra­molecular features   

**Figure 2:**
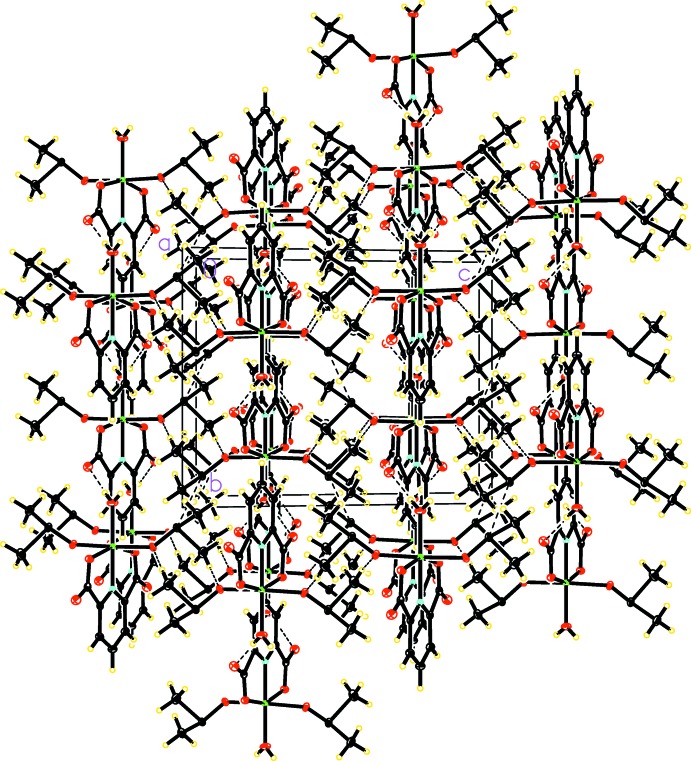
The crystal packing of the title compound, showing hydrogen bonds as dashed lines.

In the crystal, the mononuclear complexes are linked *via* an extensive network of C—H⋯O and O—H⋯O hydrogen bonds where the hydrogen-bond donors are the C—H groups of DMSO mol­ecules and the O—H groups of the coordinating water mol­ecules and the hydrogen-bond acceptors are the non-coordinating O2 atoms of the 2,6-pyridine di­carb­oxy­lic acid dianion and the O4 atoms of the DMSO mol­ecules (Table 1 ▸, Fig. 2 ▸). In the crystal packing, layers of the Ni^II^–dpa^2−^ complexes alternating with layers of DMSO mol­ecules are formed parallel to (001) (Fig. 2 ▸).

## Database survey   

A search of the Cambridge Structural Database (Groom *et al.*, 2016 ▸) returned eight structures that are related to the title complex. These structures incorporate some or all of the ligands in the title complex and include mononuclear and binuclear complexes, as well as coordination polymers. One of the structures is *mer*-aqua-bis­(di­methyl­sulfoxide-*O*)(pyridine-2,6-di­carboxyl­ato-*N,O,O′*)cobalt(II) (Felloni *et al.*, 2010 ▸) that crystallizes isotypically with the title complex. Therefore bond lengths and bond angles surrounding the Co^II^ are very similar to those in the title complex. Another mononuclear complex is aqua­chlorido­(dimethyl sulfoxide-*O*)(pyridine-2,6-di­carboxyl­ato-*N,O,O′*)iron(III) (Rafizadeh *et al.*, 2006 ▸). In the crystal, this complex also forms alternating layers parallel to (001) due to the inter­digitation of DMSO mol­ecules. Other complexes in the search results involve either coordinating or non-coordinating DMSO mol­ecules and one or more 2,6-pyridine di­carboxyl­ate dianions coordinating to a metal ion.

## Synthesis and crystallization   

Anhydrous NiCO_3_ (0.33 mmol, 39.56 mg), 2,6-pyridine di­carb­oxy­lic acid (0.33 mmol, 55.71 mg), and 2-chloro­pyrazine (0.50 mmol, 57.26 mg) were mixed in 10 ml dimethyl sulfoxide under stirring for 30 minutes. The resulting mixture was placed in a stainless steel autoclave. The autoclave was then sealed and heated to 373 K for 24 h and cooled to room temperature at a rate of 0.1 K per minute. The resulting green crystals were collected by filtration (yield 30.0%). Selected IR bands (KBr, cm^−1^): 3134.9 (O—H), 1613.1 (C=O), 1365.8 (C—O), 999.6 (S=O).

## Refinement   

Crystal data, data collection and structure refinement details are summarized in Table 2 ▸. The title complex is located on a twofold rotation axis, thus half of it occupies the asymmetric unit. The coordinating water mol­ecule lies on the symmetry axis which requires one hydrogen atom to be located while the other is related by symmetry. This hydrogen atom was obtained from a difference-Fourier map and was refined freely. The other hydrogen atoms were positioned geometrically (C—H = 0.93/1.00 Å) and allowed to ride with *U*_iso_(H)= 1.2/1.5*U*_eq_(C). Methyl hydrogen atoms were allowed to rotate but not to tip.

## Supplementary Material

Crystal structure: contains datablock(s) I. DOI: 10.1107/S2056989017006090/wm5380sup1.cif

Structure factors: contains datablock(s) I. DOI: 10.1107/S2056989017006090/wm5380Isup2.hkl

CCDC reference: 1545417

Additional supporting information:  crystallographic information; 3D view; checkCIF report

## Figures and Tables

**Table 1:** Hydrogen-bond geometry (Å, °)

*D*—H⋯*A*	*D*—H	H⋯*A*	*D*⋯*A*	*D*—H⋯*A*
O3—H3⋯O2^i^	0.790 (17)	1.867 (17)	2.6555 (10)	176.3 (18)
C5—H5*B*⋯O2^i^	0.98	2.58	3.3650 (14)	137
C6—H6*A*⋯O2^ii^	0.98	2.62	3.3504 (13)	132
C6—H6*B*⋯O4^iii^	0.98	2.38	3.3302 (14)	162

Symmetry codes: (i) 
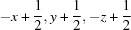
; (ii) 
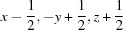
; (iii) 
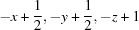
.

**Table 2:** Experimental details

Crystal data	
Chemical formula	[Ni(C_7_H_3_NO_4_)(C_2_H_6_OS)_2_(H_2_O)]
*M*_r_	398.08
Crystal system, space group	Monoclinic, *C*2/*c*
Temperature (K)	100
*a*, *b*, *c* (Å)	9.8767 (5), 11.4597 (5), 14.3166 (7)
β (°)	104.4577 (7)
*V* (Å^3^)	1569.09 (13)
*Z*	4
Radiation type	Mo *K*α
μ (mm^−1^)	1.53
Crystal size (mm)	0.46 × 0.17 × 0.11

Data collection	
Diffractometer	Bruker APEXII DUO CCD
Absorption correction	Multi-scan (*SADABS*; Bruker, 2014 ▸)
*T*_min_, *T*_max_	0.678, 0.910
No. of measured, independent and observed [*I* > 2σ(*I*)] reflections	18746, 1936, 1907
*R*_int_	0.011
(sin θ/λ)_max_ (Å^−1^)	0.666

Refinement	
*R*[*F* ^2^ > 2σ(*F* ^2^)], *wR*(*F* ^2^), *S*	0.016, 0.043, 1.06
No. of reflections	1936
No. of parameters	108
H-atom treatment	H atoms treated by a mixture of independent and constrained refinement
Δρ_max_, Δρ_min_ (e Å^−3^)	0.46, −0.24

Computer programs: *APEX2* and *SAINT* (Bruker, 2014 ▸), *SHELXT* (Sheldrick, 2015*a* ▸), *SHELXL2014* (Sheldrick, 2015*b* ▸), *XP* in *SHELXTL-Plus* (Sheldrick, 2008 ▸) and *publCIF* (Westrip, 2010 ▸).

## supplementary crystallographic information

### Crystal data

**Table:**

[Ni(C_7_H_3_NO_4_)(C_2_H_6_OS)_2_(H_2_O)]	*F*(000) = 824
*M**_r_* = 398.08	*D*_x_ = 1.685 Mg m^−^^3^
Monoclinic, *C*2/*c*	Mo *K*α radiation, λ = 0.71073 Å
*a* = 9.8767 (5) Å	Cell parameters from 9953 reflections
*b* = 11.4597 (5) Å	θ = 2.0–28.0°
*c* = 14.3166 (7) Å	µ = 1.53 mm^−^^1^
β = 104.4577 (7)°	*T* = 100 K
*V* = 1569.09 (13) Å^3^	Block, green
*Z* = 4	0.46 × 0.17 × 0.11 mm

### Data collection

**Table:**

Bruker APEXII DUO CCD diffractometer	1907 reflections with *I* > 2σ(*I*)
Radiation source: fine-focus sealed tube	*R*_int_ = 0.011
phi and ω scans	θ_max_ = 28.3°, θ_min_ = 2.8°
Absorption correction: multi-scan (*SADABS*; Bruker, 2014)	*h* = −13→13
*T*_min_ = 0.678, *T*_max_ = 0.910	*k* = −15→15
18746 measured reflections	*l* = −19→19
1936 independent reflections	

### Refinement

**Table:**

Refinement on *F*^2^	Primary atom site location: structure-invariant direct methods
Least-squares matrix: full	Secondary atom site location: difference Fourier map
*R*[*F*^2^ > 2σ(*F*^2^)] = 0.016	Hydrogen site location: mixed
*wR*(*F*^2^) = 0.043	H atoms treated by a mixture of independent and constrained refinement
*S* = 1.06	*w* = 1/[σ^2^(*F*_o_^2^) + (0.0225*P*)^2^ + 1.5829*P*] where *P* = (*F*_o_^2^ + 2*F*_c_^2^)/3
1936 reflections	(Δ/σ)_max_ = 0.001
108 parameters	Δρ_max_ = 0.46 e Å^−^^3^
0 restraints	Δρ_min_ = −0.24 e Å^−^^3^

### Special details

**Table:**

Geometry. All esds (except the esd in the dihedral angle between two l.s. planes) are estimated using the full covariance matrix. The cell esds are taken into account individually in the estimation of esds in distances, angles and torsion angles; correlations between esds in cell parameters are only used when they are defined by crystal symmetry. An approximate (isotropic) treatment of cell esds is used for estimating esds involving l.s. planes.
Refinement. All H atoms were positioned geometrically ( C—H = 0.93/1.00 Å) and allowed to ride with *U*_iso_(H)= 1.2/1.5*U*_eq_(C). Methyl ones were allowed to rotate around the corresponding C—C. The Ni complex is located on a 2-fold rotational axis of symmetry thus half of it occupies the asymmetric unit. The coordinated water molecule lies on the symmetry axis which requires one proton to be located while the other is related by the symmetry. The water proton was obtained from a Difference Fourier map and refined freely.

### Fractional atomic coordinates and isotropic or equivalent isotropic displacement parameters (Å^2^)

**Table:**

	*x*	*y*	*z*	*U*_iso_*/*U*_eq_	
Ni1	0.0000	0.32387 (2)	0.2500	0.00867 (6)	
S1	0.08818 (3)	0.40008 (2)	0.46249 (2)	0.01346 (7)	
O1	0.16898 (7)	0.28806 (6)	0.18844 (5)	0.01235 (14)	
O2	0.29613 (8)	0.13796 (7)	0.15626 (6)	0.01647 (15)	
O3	0.0000	0.49874 (10)	0.2500	0.0186 (2)	
H3	0.0626 (17)	0.5376 (15)	0.2789 (12)	0.033 (4)*	
O4	0.13764 (8)	0.32772 (6)	0.38736 (5)	0.01257 (14)	
N1	0.0000	0.15272 (10)	0.2500	0.0095 (2)	
C1	0.0000	−0.08533 (12)	0.2500	0.0151 (3)	
H1A	0.0000	−0.1682	0.2500	0.018*	
C2	0.10055 (10)	−0.02463 (9)	0.21553 (7)	0.01328 (18)	
H2A	0.1691	−0.0650	0.1919	0.016*	
C3	0.09697 (10)	0.09684 (8)	0.21703 (7)	0.01026 (17)	
C4	0.19693 (10)	0.18040 (9)	0.18444 (7)	0.01114 (18)	
C5	0.24415 (12)	0.46192 (10)	0.53687 (8)	0.0204 (2)	
H5A	0.2213	0.5058	0.5897	0.031*	
H5B	0.2865	0.5145	0.4982	0.031*	
H5C	0.3102	0.3994	0.5636	0.031*	
C6	0.04879 (12)	0.29765 (11)	0.54609 (8)	0.0213 (2)	
H6A	0.0278	0.3397	0.6004	0.032*	
H6B	0.1294	0.2463	0.5699	0.032*	
H6C	−0.0324	0.2508	0.5138	0.032*	

### Atomic displacement parameters (Å^2^)

**Table:**

	*U*^11^	*U*^22^	*U*^33^	*U*^12^	*U*^13^	*U*^23^
Ni1	0.00926 (9)	0.00735 (9)	0.00927 (9)	0.000	0.00206 (6)	0.000
S1	0.01554 (12)	0.01458 (12)	0.00942 (12)	0.00621 (9)	0.00155 (9)	−0.00145 (8)
O1	0.0122 (3)	0.0109 (3)	0.0147 (3)	−0.0010 (3)	0.0048 (3)	−0.0004 (3)
O2	0.0138 (3)	0.0181 (4)	0.0196 (4)	0.0040 (3)	0.0081 (3)	0.0023 (3)
O3	0.0178 (5)	0.0085 (5)	0.0237 (6)	0.000	−0.0057 (4)	0.000
O4	0.0131 (3)	0.0143 (3)	0.0099 (3)	0.0037 (3)	0.0021 (3)	−0.0030 (2)
N1	0.0102 (5)	0.0094 (5)	0.0083 (5)	0.000	0.0011 (4)	0.000
C1	0.0189 (7)	0.0091 (6)	0.0170 (7)	0.000	0.0038 (5)	0.000
C2	0.0143 (4)	0.0116 (4)	0.0136 (4)	0.0025 (4)	0.0028 (3)	−0.0008 (3)
C3	0.0103 (4)	0.0114 (4)	0.0085 (4)	0.0005 (3)	0.0012 (3)	0.0001 (3)
C4	0.0099 (4)	0.0140 (5)	0.0088 (4)	−0.0002 (3)	0.0009 (3)	0.0006 (3)
C5	0.0233 (5)	0.0204 (5)	0.0137 (5)	0.0012 (4)	−0.0022 (4)	−0.0044 (4)
C6	0.0227 (5)	0.0259 (6)	0.0180 (5)	0.0069 (4)	0.0105 (4)	0.0038 (4)

### Geometric parameters (Å, º)

**Table:**

Ni1—N1	1.9613 (12)	N1—C3^i^	1.3327 (11)
Ni1—O3	2.0040 (11)	C1—C2^i^	1.3994 (12)
Ni1—O4	2.0934 (7)	C1—C2	1.3995 (12)
Ni1—O4^i^	2.0934 (7)	C1—H1A	0.9500
Ni1—O1^i^	2.1130 (7)	C2—C3	1.3927 (14)
Ni1—O1	2.1130 (7)	C2—H2A	0.9500
S1—O4	1.5316 (7)	C3—C4	1.5296 (13)
S1—C5	1.7860 (11)	C5—H5A	0.9800
S1—C6	1.7872 (12)	C5—H5B	0.9800
O1—C4	1.2686 (12)	C5—H5C	0.9800
O2—C4	1.2476 (12)	C6—H6A	0.9800
O3—H3	0.790 (17)	C6—H6B	0.9800
N1—C3	1.3326 (11)	C6—H6C	0.9800
			
N1—Ni1—O3	180.0	C2^i^—C1—C2	120.38 (13)
N1—Ni1—O4	91.21 (2)	C2^i^—C1—H1A	119.8
O3—Ni1—O4	88.79 (2)	C2—C1—H1A	119.8
N1—Ni1—O4^i^	91.21 (2)	C3—C2—C1	117.93 (10)
O3—Ni1—O4^i^	88.79 (2)	C3—C2—H2A	121.0
O4—Ni1—O4^i^	177.58 (4)	C1—C2—H2A	121.0
N1—Ni1—O1^i^	78.80 (2)	N1—C3—C2	120.60 (9)
O3—Ni1—O1^i^	101.20 (2)	N1—C3—C4	112.51 (9)
O4—Ni1—O1^i^	90.43 (3)	C2—C3—C4	126.88 (9)
O4^i^—Ni1—O1^i^	90.04 (3)	O2—C4—O1	126.23 (9)
N1—Ni1—O1	78.80 (2)	O2—C4—C3	118.26 (9)
O3—Ni1—O1	101.20 (2)	O1—C4—C3	115.51 (9)
O4—Ni1—O1	90.04 (3)	S1—C5—H5A	109.5
O4^i^—Ni1—O1	90.43 (3)	S1—C5—H5B	109.5
O1^i^—Ni1—O1	157.60 (4)	H5A—C5—H5B	109.5
O4—S1—C5	104.76 (5)	S1—C5—H5C	109.5
O4—S1—C6	106.00 (5)	H5A—C5—H5C	109.5
C5—S1—C6	99.26 (6)	H5B—C5—H5C	109.5
C4—O1—Ni1	114.28 (6)	S1—C6—H6A	109.5
Ni1—O3—H3	124.3 (13)	S1—C6—H6B	109.5
S1—O4—Ni1	115.15 (4)	H6A—C6—H6B	109.5
C3—N1—C3^i^	122.56 (12)	S1—C6—H6C	109.5
C3—N1—Ni1	118.72 (6)	H6A—C6—H6C	109.5
C3^i^—N1—Ni1	118.72 (6)	H6B—C6—H6C	109.5

Symmetry code: (i) −*x*, *y*, −*z*+1/2.

### Hydrogen-bond geometry (Å, º)

**Table:**

*D*—H···*A*	*D*—H	H···*A*	*D*···*A*	*D*—H···*A*
O3—H3···O2^ii^	0.790 (17)	1.867 (17)	2.6555 (10)	176.3 (18)
C5—H5*B*···O2^ii^	0.98	2.58	3.3650 (14)	137
C6—H6*A*···O2^iii^	0.98	2.62	3.3504 (13)	132
C6—H6*B*···O4^iv^	0.98	2.38	3.3302 (14)	162

Symmetry codes: (ii) −*x*+1/2, *y*+1/2, −*z*+1/2; (iii) *x*−1/2, −*y*+1/2, *z*+1/2; (iv) −*x*+1/2, −*y*+1/2, −*z*+1.

## References

[bb1] Bruker (2014). *APEX2*, *SAINT* and *SADABS*. Bruker Inc., Madison, Wisconsin, USA.

[bb2] Cook, T. R., Zheng, Y. R. & Stang, P. J. (2013). *Chem. Rev.* **113**, 734–777. 10.1021/cr3002824 PMC3764682 23121121

[bb3] Du, M., Li, C. P., Liu, C. S. & Fang, S. M. (2013). *Coord. Chem. Rev.* **257**, 1282–1305.

[bb4] Felloni, M., Blake, A. J., Hubberstey, P., Teat, S. J., Wilson, C. & Schröder, M. (2010). *CrystEngComm*, **12**, 1576–1589.

[bb5] Groom, C. R., Bruno, I. J., Lightfoot, M. P. & Ward, S. C. (2016). *Acta Cryst.* B**72**, 171–179. 10.1107/S2052520616003954 PMC4822653 27048719

[bb6] Li, C. P. & Du, M. (2011). *Chem. Commun.* **47**, 5958–5972. 10.1039/c1cc10935a 21475759

[bb7] Liu, C., Thuijs, A. E., Felts, A. C., Ballouk, H. F. & Abboud, K. A. (2016). *Acta Cryst.* E**72**, 768–771. 10.1107/S2056989016007064 PMC4908528 27308038

[bb8] Rafizadeh, M., Mehrabi, B. & Amani, V. (2006). *Acta Cryst.* E**62**, m1332–m1334.

[bb9] Robin, A. Y. & Fromm, K. M. (2006). *Coord. Chem. Rev.* **250**, 2127–2157.

[bb10] Sheldrick, G. M. (2008). *Acta Cryst.* A**64**, 112–122. 10.1107/S0108767307043930 18156677

[bb11] Sheldrick, G. M. (2015*a*). *Acta Cryst.* A**71**, 3–8.

[bb12] Sheldrick, G. M. (2015*b*). *Acta Cryst.* C**71**, 3–8.

[bb13] Wang, C., Liu, D. & Lin, W. (2013). *J. Am. Chem. Soc.* **135**, 13222–13234. 10.1021/ja308229p PMC3800686 23944646

[bb14] Westrip, S. P. (2010). *J. Appl. Cryst.* **43**, 920–925.

